# Attitudes and perceptions of outpatients towards adoption of telemedicine in healthcare during COVID-19 pandemic

**DOI:** 10.1007/s11845-021-02729-6

**Published:** 2021-08-16

**Authors:** Nithesh Naik, Sufyan Ibrahim, Sumedha Sircar, Vathsala Patil, Belthangady Monu Zeeshan Hameed, Bhavan Prasad Rai, Piotr Chłosta, Bhaskar K. Somani

**Affiliations:** 1 Department of Mechanical and Manufacturing Engineering, Manipal Institute of Technology, Manipal Academy of Higher Education, Manipal, Karnataka India; 2 iTRUE (International Training and Research in Uro-Oncology and Endourology) Group, Manipal, Karnataka India; 3 Kasturba Medical College of Manipal, Manipal Academy of Higher Education, Manipal, Karnataka India; 4 Department of Oral Medicine and Radiology, Manipal College of Dental Sciences, Manipal, Manipal Academy of Higher Education, Manipal, Karnataka India; 5 Department of Urology, Father Muller Medical College, Mangalore, Karnataka India; 6 Department of Urology, Freeman Hospital, Newcastle, UK; 7 Department of Urology, Jagiellonian University in Krakow, Kraków, Poland; 8 Department of Urology, University Hospital Southampton NHS Trust, Southampton, UK

**Keywords:** COVID-19, Healthcare delivery, Perception, Telehealth, Telemedicine

## Abstract

**Background:**

Asia is home to a burgeoning market for telemedicine with the availability of cheaper smartphones and internet services. Due to a rise in telemedicine use by doctors and patients, it is imperative to understand the perception of patients towards the adoption of telemedicine, the availability of telemedicine to the general population, the frequency with which patients avail these services, and the motivation or apprehensions in using them, especially during the COVID-19 pandemic.

**Aims:**

The study is performed to understand the behavioral attitude and perceptions of the population regarding telemedicine and, in doing so, make services more user-friendly for patients.

**Methods:**

A total of 1170 participants were surveyed using a structured online questionnaire to assess the perceptions towards the adoption of telemedicine in healthcare delivery services. Multivariate analysis was performed to identify key variables of knowledge and attitude affecting the utilization of telemedicine.

**Results:**

Of the total respondents, 35.3% of patients never encountered telemedicine before and 26.9% did not come across telemedicine even during the COVID-19 pandemic.

**Conclusion:**

Understanding the perceptions of patients, using targeted health education, positive communication, and behavioral modifications, is the key factor to be addressed to mitigate the apprehensions towards telemedicine and improve the utilization of the services.

## Introduction

Telemedicine has been defined by the WHO as “The delivery of health care services by the healthcare professionals using information and communication technologies.” The technology enables the exchange of valid information for the diagnosis, treatment, and prevention of diseases. It also provides a platform for research and continuing education of healthcare service providers [1]. Asia is the home to a burgeoning market for telemedicine services due to ease of availability, accessibility of affordable internet, better bandwidth, and smartphones with video cameras. The COVID-19 pandemic has garnered the relevance of telemedicine and drawn the highest attention ever since its advent [2, 3]. Telemedicine has played a potential role in times of humanitarian crisis like hurricanes and pandemics wherein victims were relocated from their homes and their primary healthcare providers [4, 5]. It is also playing a crucial role in the present pandemic, as one of the safest interactive means between the patients and clinicians as social distancing is necessary [6]. Southeast Asian countries like Myanmar, Sri Lanka, Indonesia, Bangladesh, and India have issued legislation for telemedicine implementation. They have also witnessed an increase in the uptake among patients and providers. However, to scale up its usage and maintain long-term sustainability, a user-friendly and robust system needs to be adapted by analyzing evidence about telemedicine and its use among patients and health professionals [7]. The few advantages of telemedicine are that it offers healthcare services to people in rural or poorly accessible areas, elderly populations with mobility issues, and vulnerable patients from unnecessary exposure to the infectious environment of hospitals [6, 8]. Less or no transportation and waiting time, easy access to specialists, lower risk of catching a new infection, and better healthcare management are some added benefits of telemedicine to patients and healthcare professionals. It also facilitates frequent consultation with their physicians, ensuring better management of medication, lifestyle, and health complications without having the difficulties of going into hospitals [8–10].

However, availing of telemedicine can also be a challenging task for the elderly, vulnerable populations as it requires good knowledge of the technology and gadgets for effective communication [11, 12]. In telemedicine, lack of physical examination can result in patient apprehension and potentially impede the patient-doctor rapport. Breach of patient confidentiality and private medical data are few concerns associated with the usage of telemedicine [9, 13]. Few companies deny insurance coverage for telemedicine consultations, and there is no clear medico-legal framework or regulatory body regarding telemedicine used to resolve issues like privacy, malpractice, and liabilities. These can lead to underconfidence and lack of trust in using telemedicine [14–17]. With the emergence of the COVID-19 pandemic, most of Southeast Asian countries have altered policies to reduce these barriers to telemedicine and expand mobile health expansibility [7].

Lack of awareness and training about telemedicine among undergraduates and postgraduates has partly been responsible for doctors’ hesitation to adopt the technology [18]. However, with the rise of demand and use of telemedicine, it is imperative to understand the perception of patients towards the adoption of telemedicine during the present COVID-19 pandemic. With the current situation of the pandemic, there is a need for greater awareness about telemedicine among the population. Therefore, this study was performed to understand the attitude and perception of outpatients with regard to telemedicine, to make it user-friendly for patients in the future.

## Materials and methods

### Study design and data collection

A cross-sectional study was carried out from November 2020 to December 2020 through a web-based survey on the general population outpatient. Individuals above the age group of 18 years, who had hospital outpatient experience, with and/or without telemedicine experience, able to navigate Google Forms, and willing to fill in the questionnaire were included in the study. The study was approved by the Institutional Ethics Committee (Reference No. IEC 612/2020).

A survey tool was constructed to determine the outpatient’s behavioral attitudes and perceptions about telemedicine during the COVID-19 pandemic. The survey was disseminated primarily via mailing lists and social media platforms like Facebook, Instagram, WhatsApp, and Twitter. The first invitation to participate in the study was sent out on November 6, 2020. A reminder was sent after 2 weeks of the initial invite and finally concluded on December 5, 2020. A total of 1170 participants who responded to the survey were recruited for the study based on the snowball sampling technique, where each respondent were motivated to refer links to their contacts for participation. The participants were auto-directed to the survey by clicking the link, and the participation was voluntary and no incentives were rewarded.

### Survey design

The primary objective was to assess the attitude and perceptions of the outpatients towards the adoption of telemedicine during the present pandemic, in comparison to pre-pandemic times. The secondary objective was to identify the concerns and understand the patient’s perspectives on the factors that may hinder the usage of telemedicine in healthcare practices. The structured online questionnaire-based survey consisting of thirty questions in total were asked in English via Google Forms. The information related to socio-demographics, attitudes, perceptions, and practices towards telemedicine services was collected.

### Statistical analysis

All data were collected within the Google Forms spreadsheet system, and only the study investigators could access these data. Statistical data analysis was performed using SPSS (version 26) software. Frequencies and percentages through descriptive analysis of all the answers were described. Mean with 95% confidence interval was calculated to quantify different variables of attitude towards telemedicine. At less than 0.05 *P*-value, statistical significance was set in all tests.

## Results

A total of 1170 responses were collected with a majority of male respondents (52.2%). The maximum response rate was from the participants in the age group of 25–34 years (43.8%) followed by people aged 18–24 years (30.4%). The most elderly group, designated as those above 55 years of age, had the least representation (1.9%). Respondents’ socio-demographic characteristics are presented in Table 1. In the overall response, 35.3% of patients never encountered telemedicine before COVID-19 and 26.9% did not avail telemedicine even during the COVID-19 pandemic as shown in Table 1. Among those who availed telemedicine services, 48.0% of them opted for consultation and 34.2% for booking an appointment as their primary reasons.

**Table 1:** Socio-demographic characteristics of the study participants

**Socio-demographic characteristics**	***N* (%)**
***Gender***	
Male	610 (52.2)
Female	559 (47.8)
***Age***	
18–24	356 (30.4)
25–34	512 (43.8)
35–44	217 (18.5)
45–54	62 (5.4)
> 55	23 (1.9)
**Perception of patients towards telemedicine**	
***People availing telemedicine before COVID-19***	
Frequently	187 (15.9)
Never	412 (35.3)
Occasionally	220 (18.8)
Rarely	351 (30)
***People availing telemedicine during COVID-19***	
Frequently	246 (21.0)
Never	315 (26.9)
Occasionally	310 (26.5)
Rarely	299 (25.6)
***Purpose of availing telemedicine facility***	
Booking an appointment	400 (34.2)
Consultation	562 (48.0)
Treatment	368 (31.5)
Review of the doctors	371 (31.7)

The attitude/perception of the participants availing (*N* = 758) and those who never used (*N* = 412) telemedicine in pre-COVID-19 is summarized in Table 2. The perception of the participants who used telemedicine pre-COVID-19 showed an overall score of more than 3, indicating a neutral to positive attitude towards it. Convenience facility (mean score = 3.15), scheduling an appointment (mean score = 3.14), and immediate response from a specialist (mean score = 3.14) were highly scored by the participants; however, a few of them felt that telemedicine is more approachable and useful to rural people pre-COVID-19 (mean score = 2.94). The scores of the participants who had not used telemedicine pre-COVID-19 were lesser (≤ 3) compared to those of the ones who had used telemedicine, indicating a neutral to negative attitude. Scheduling appointments, opinion of more than one doctor, and the information provided by doctors as reliable were scored with a mean score of 2.98, 2.94, and 2.9, respectively, by the participants.

**Table 2:** Perception/attitude of participants towards telemedicine in pre-COVID-19

**Reason to influence for the telemedicine**	**Strongly disagree** ***N* (%)**	**Disagree** ***N* (%)**	**Neutral** ***N* (%)**	**Agree** ***N* (%)**	**Strongly agree** ***N* (%)**	**Mean**
**The attitude of the people who have used telemedicine pre-COVID-19 (*N* = 758)**						
Consumes less time in medical consultation and treatment	92 (12.1)	165 (21.8)	211 (27.8)	130 (17.2)	160 (21.1)	3.13
Cost effective	81 (10.7)	199 (26.3)	205 (27.0)	137 (18.1)	136 (17.9)	3.06
Convenience facility	81 (10.7)	171 (22.6)	208 (27.4)	148 (19.5)	150 (19.8)	3.15
Scheduling appointments and talking to healthcare providers become easy	80 (10.6)	182 (24.0)	197 (26.0)	153 (20.2)	146 (19.2)	3.14
Immediate response from specialist	77 (10.2)	174 (22.9)	218 (28.8)	147 (19.4)	142 (18.7)	3.14
Follow-up and procedure become more convenient	78 (10.3)	183 (24.1)	199 (26.3)	151 (19.9)	147 (19.4)	3.11
Opinion of more than one doctor is possible	78 (10.3)	190 (25.0)	205 (27.0)	137 (18.1)	148 (19.5)	3.01
There is no insurance or reimbursement problem involved in usage of telemedicine facility	106 (14.0)	177 (23.3)	201 (26.5)	152 (20.1)	122 (16.1)	3.13
Information provided by doctors is reliable	67 (8.8)	203 (26.8)	194 (25.6)	156 (20.6)	138 (18.2)	3.14
Telemedicine helps in reducing unnecessary visits to hospitals	94 (12.4)	160 (21.1)	201 (26.5)	149 (19.7)	154 (20.3)	3.07
Providing 24 × 7 access to specialist	104 (13.7)	174 (23.0)	185 (24.4)	153 (20.2)	142 (18.7)	3.06
Telemedicine is more approachable and useful to rural people	93 (12.3)	187 (24.7)	193 (25.5)	152 (20.1)	133 (17.5)	2.95
**The attitude of the people who have never used telemedicine pre-COVID-19 (*N* = 412)**						
Scheduling appointments and talking to healthcare providers become easy	76 (18.4)	82 (19.9)	115 (27.9)	51 (12.4)	88 (21.4)	2.98
Immediate response from specialist	70 (17.0)	88 (21.4)	111 (26.9)	54 (13.1)	89 (21.6)	3.01
Follow-up and procedure become more convenient	64 (15.5)	96 (23.3)	111 (26.9)	58 (14.1)	83 (20.1)	3.00
Opinion of more than one doctor is possible	79 (19.2)	87 (21.1)	109 (26.5)	52 (12.6)	85 (20.6)	2.94
Information provided by doctors is reliable	74 (18.0)	98 (23.8)	110 (26.7)	54 (13.1)	76 (18.4)	2.90
Telemedicine is more approachable and useful to rural people	78 (18.9)	77 (18.7)	112 (27.2)	57 (13.8)	88 (21.4)	3.00

The attitude/perception of the participants who are availing (*N* = 855) and never used (*N* = 315) telemedicine during COVID-19 is summarized in Table 3. The perception of the participants who used telemedicine during COVID-19 showed an overall score greater than 3, indicating a positive attitude towards it. Convenience facility (mean score = 3.13), scheduling an appointment (mean score = 3.12), and immediate response from a specialist (mean score = 3.13) were highly scored by the participants. However, telemedicine being more approachable and useful to rural people during COVID-19 was rated equally (mean score = 3.05) among people who have used and not used telemedicine. The scores of the participants who had not used telemedicine during COVID-19 were greater (≥ 3.05), indicating a positive attitude towards its usage and acceptance. Scheduling appointments, opinion of more than one doctor, and the information provided by doctors as reliable were scored with a mean score of 3.12, 3.09, and 3.09, respectively, by the participants. Overall, the outpatient respondents in the study showed a positive attitude towards the adoption of telemedicine services during COVID-19.

**Table 3:** Attitude of participant outpatients towards telemedicine during COVID-19

**Reason to influence for the telemedicine**	**Strongly disagree** ***N* (%)**	**Disagree** ***N* (%)**	**Neutral** ***N* (%)**	**Agree** ***N* (%)**	**Strongly agree** ***N* (%)**	**Mean**
**The attitude of the patients who have used telemedicine during COVID-19 (*N* = 855)**						
Consumes less time in medical consultation and treatment	122 (14.3)	184 (21.5)	220 (25.7)	146 (17.1)	183 (21.4)	3.10
Cost effective	107 (12.5)	222 (26.0)	222 (26.0)	150 (17.5)	154 (18.0)	3.03
Convenience facility	105 (12.3)	186 (21.8)	233 (27.3)	157 (18.4)	174 (20.4)	3.13
Scheduling appointments and talking to healthcare providers become easy	107 (12.5)	193 (22.6)	218 (25.5)	164 (19.2)	173 (20.2)	3.12
Immediate response from specialist	98 (11.5)	190 (22.2)	236 (27.6)	165 (19.3)	166 (19.4)	3.13
Follow-up and procedure become more convenient	97 (11.3)	206 (24.1)	216 (25.3)	167 (19.5)	169 (19.8)	3.13
Opinion of more than one doctor is possible	106 (12.4)	201 (23.5)	224 (26.2)	157 (18.4)	167 (19.5)	3.09
There is no insurance or reimbursement problem involved in usage of telemedicine facility	130 (15.2)	200 (23.4)	212 (24.8)	172 (20.1)	141 (16.5)	2.99
Information provided by doctors is reliable	93 (10.9)	217 (25.4)	216 (25.3)	174 (20.4)	155 (18.1)	3.09
Telemedicine helps in reducing unnecessary visits to hospitals	123 (14.4)	166 (19.4)	219 (25.6)	170 (19.9)	177 (20.7)	3.13
Providing 24 × 7 access to specialist	135 (15.8)	194 (22.7)	195 (22.8)	173 (20.2)	158 (18.5)	3.03
Telemedicine is more approachable and useful to rural people	124 (14.5)	198 (23.2)	205 (24.0)	170 (19.9)	158 (18.5)	3.05
**The attitude of the patient who has never used telemedicine during COVID-19 (*N* = 315)**						
Scheduling appointments and talking to healthcare providers become easy	49 (15.6)	71 (22.5)	94 (29.8)	40 (12.7)	61 (19.4)	3.12
Immediate response from specialist	49 (15.6)	72 (22.9)	93 (29.5)	36 (11.4)	65 (20.6)	3.13
Follow-up and procedure become more convenient	45 (14.3)	73 (23.2)	94 (29.8)	42 (13.3)	61 (19.4)	3.12
Opinion of more than one doctor is possible	51 (16.2)	76 (24.1)	90 (28.6)	32 (10.2)	66 (21.0)	3.09
Information provided by doctors is reliable	48 (15.2)	84 (26.7)	88 (27.9)	36 (11.4)	59 (18.7)	3.09
Telemedicine is more approachable and useful to rural people	58 (18.4)	73 (23.2)	89 (28.3)	28 (8.9)	67 (21.3)	3.05

The experience of the participants using telemedicine services pre-COVID-19 (*N* = 758) was ranked using the Likert scale with most preferable as 1 to least preferable as 5 (Table 4). The overall experience was positive (1–3) for all domains like “usage of telemedicine is economical” (69.2%), “qualitative care” (68.5%), “easy transfer of medical reports, X-rays, and availability of doctors” (68.2%), “waiting time to meet consultant is reduced” (68.0%), and “the treatment offered through telemedicine is the same as that of hospital” (68.4%).

**Table 4:** Experience of participants using telemedicine services pre-COVID (*N* = 758)

**Ranking from 1 as most preferred to 5 as least preferred**	**(1)** ***N* (%)**	**(2)** ***N* (%)**	**(3)** ***N* (%)**	**(4)** ***N* (%)**	**(5)** ***N* (%)**
Usage of telemedicine is economical	128 (16.9)	196 (25.9)	200 (26.4)	148 (19.5)	86 (11.3)
Qualitative care	102 (13.5)	205 (27.0)	212 (28.0)	155 (20.4)	84 (11.1)
Easy transfer of medical reports, X-rays, and availability of doctors	129 (17.0)	171 (22.6)	217 (28.7)	155 (20.4)	86 (11.3)
Waiting time to meet consultation is reduced	136 (17.95)	182 (24.0)	198 (26.1)	142 (18.75)	100 (13.2)
The treatment offered through telemedicine is the same as that of hospital	118 (15.55)	168 (22.15)	232 (30.6)	146 (19.3)	94 (12.4)

The experience of the participants using telemedicine during COVID-19 (*N* = 855) was ranked using the Likert scale with most preferable as 1 to least preferable as 5 (Table 5). In this category also, the overall experience was positive (1–3) for all domains like “usage of telemedicine is economical” (69.2%), “qualitative care” (69.3%), “easy transfer of medical reports, X-rays, and availability of doctors” (68.7%), “waiting time to meet consultant is reduced” (68.3%), and “the treatment offered through telemedicine is the same as that of hospital” (69.6%).

**Table 5:** Experience of participants using telemedicine services during COVID-19 (*N* = 855)

**Ranking from 1 as most preferred to 5 as least preferred**	**(1)** ***N* (%)**	**(2)** ***N* (%)**	**(3)** ***N* (%)**	**(4)** ***N* (%)**	**(5)** ***N* (%)**
Usage of telemedicine is economical	162 (18.9)	203 (23.7)	233(27.3)	154 (18.0)	103 (12.0)
Qualitative care	129 (15.1)	230 (26.9)	233 (27.3)	166 (19.4)	97 (11.3)
Easy transfer of medical reports, X-rays, and availability of doctors	161 (18.8)	185 (21.6)	242 (28.3)	169 (19.8)	98 (11.5)
Waiting time to meet consultation is reduced	166 (19.4)	199 (23.3)	219 (25.6)	153 (17.9)	118 (13.8)
The treatment offered through telemedicine is the same as that of hospital	146 (17.1)	189 (22.1)	260 (30.4)	164 (19.2)	96 (11.2)

**Fig. 1:**
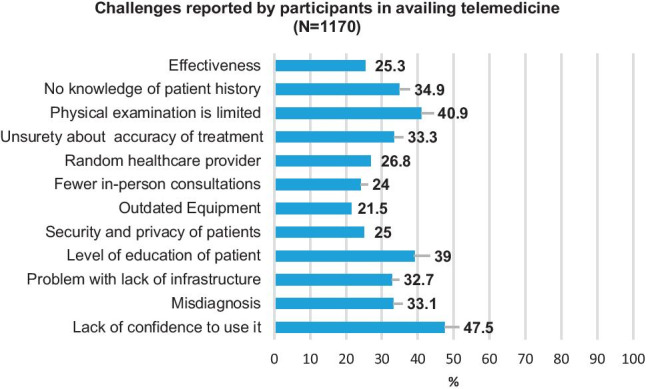
Challenges faced by the participants in availing of telemedicine services

Challenges faced by the participants (*N* = 1170) in availing telemedicine services are shown in Fig. 1. Lack of confidence in using it (47.5%), limited or no physical examination (40.9%), and the low level of their education about telemedicine (39%) influencing the ease of usage were reported as the major hurdles faced by majority of the participants of the survey.

## Discussion

COVID-19 has been a challenging test for countries’ to mitigate its spread and safeguard the citizens. In the subsequent waves of the pandemic, due to the mutant and highly virulent strains, a rapid spread is witnessed, resulting in great burden and burnout on healthcare professionals. In these circumstances, telemedicine has emerged as a convenient medium to get a grip over the situation. Telemedicine is used for providing online consultations for COVID-19 patients at home isolation and telemonitoring of their lab reports, oxygen saturation, blood pressure, and sugar levels by asking specific questions. Sensors for tracking the location of oxygen/ICU bed availability in hospitals, etc., were highly useful means implemented during these times. This study was performed to understand the perception and attitudes of outpatients towards telemedicine pre-COVID-19 and during COVID-19 times. The challenges and their experience using telemedicine have also been assessed [18–22].

The present study has shown an overall mean score of > 3 both pre-COVID-19 and during COVID-19, reflecting a neutral to positive perception of outpatients towards telemedicine. The majority of respondents considered telemedicine to have benefits like “more convenient follow-up,” “reducing unnecessary hospital visits,” and “more approachable and useful for rural people,” as they highly agreed upon these sections. There was also an increase in the usage of telemedicine during the pandemic period compared to the pre-pandemic period. A decrease in the number of participants who never availed of telemedicine during the pandemic signifies that people have started seeing telemedicine as a viable option to receive healthcare. No study till date has evaluated the perception of telemedicine during COVID-19 and pre-COVID-19 and compared the experience between users’ and non-users’ points of view in the Indian populations [8]. A study by Ronda et al. on the perception of telemedicine on diabetic patients reported that non-users of telemedicine and users of telemedicine had different perceptions, and incomplete knowledge or unawareness about the web portal and feeling of inadequacy with the use of computers were the most reported challenges faced by patients in using telemedicine [8].

Reflecting on the experiences of people when they availed telemedicine services during COVID-19, our study revealed that participants considered telemedicine an economically effective option, with 23.7% of people rating it “preferred,” 27.3% of people rating it neutral, and just 18.0% of people rating it less preferred. Regarding satisfaction with the quality of healthcare, 26.9% rated their experience positively and 27.3% of people rated their experience neutral; however, 11.3–19.4% rated it unsatisfactory. This could be a natural insight of people not trusting the ability of telemedicine to compensate for physical examination and in-person visits compared to conventional outpatient visits. Similar results were also observed in the study by Holtz wherein respondents mostly seemed to have a positive to neutral response to “reduced waiting time for consultation” and “easy transfer of medical reports, X-rays, and availability of doctors” overall [23]. The overall positive experience during COVID-19 has increased compared to the experience of telemedicine usage pre-COVID-19. A global survey during the pandemic by Scott et al. stated that during the pandemic, most of the patients opted for telemedicine as a medium for remote consultation [24].

There also seems to be mistrust in the efficacy of telemedicine. Although the use of telemedicine is rising, it is not yet ubiquitous, and barriers vary widely. Technology barriers and inadequate computer literacy are the major reasons, which still prevail in implementing telemedicine successfully. Of the participants in the present study, 47.5% reported a lack of confidence. This lack of confidence could be due to limited physical examination, fear of misdiagnosis, lack of patient education, and inadequate knowledge about the previous medical history. Participants also reported these as major challenges in using telemedicine. A systematic review by Scott et al. reported technical challenges, followed by resistance to change, cost, reimbursement issues, age of the patient, and level of education of patient as major barriers. They have suggested that policymakers have to formulate public policies at national and international levels to bridge gaps in the use of telemedicine in all geographical areas, particularly those in rural settings [12]. Many patients consider its use along with face-to-face interactions as necessary [9–12, 16].

Given the increasing importance of telemedicine at the time of the COVID-19 pandemic, the guidelines on the practice of telemedicine published in 2005 were revised in 2020 to focus on medical ethics, data privacy, confidentiality, documentation, digital records of consultation, and process of setting of fees for telemedicine. They emphasize principles of medical ethics, including professional norms for protecting patient privacy and confidentiality as per the Indian Medical Council Act [25].

In our study, the responses to “if they found reimbursement by insurance companies to be problem-free,” the majority of the participants disagreed both pre-COVID-19 and during COVID-19. However, the acceptance of it being hassle-free has increased slightly during COVID-19 compared to pre-COVID-19. The fact that insurance companies have been laid-back when it comes to updating their policies to include telemedicine, and that the rules for reimbursement are complicated and unclear, there may be a reason to drive this feeling, suggesting that more work needs to be done for telemedicine reimbursements and claims to be made more straightforward and accessible [9]. Humans, infrastructure, and institutional determinants are the factors that influence the adoption of telehealth and digital solutions [26].

The strength of the present study lies in the representation of a large number of responses regarding telemedicine and its uses, the dissemination of which is essential during such a highly dynamic COVID-19 situation. However, we also acknowledge few limitations like fewer responses from individuals older than 55 years, showing less representation from the elderly population. Responses in the study were from people who could answer the survey online and those with access to the internet and well versed with technology; hence, it may not be representative of all types of patients.

## Conclusion

This pandemic has shed light on the importance of telemedicine in the service delivery system. The results of the study showcased that majority of patients are keen to embark on the utilization of telemedicine as a safety-net approach during the pandemic. However, there are many apprehensions and challenges reported. The success of telemedicine in the future is associated with the ability to overcome these. Understanding the perceptions and preferences of the patients concerning their care and developing privacy-enforcing telehealth technologies is the foremost step. Policymakers and healthcare providers should develop guidelines to support the uptake of telemedicine efficiently. Targeted health education in conjunction with behavior change is also the key to improving the utilization of telemedicine services. Ultimately, adequate reimbursement and better insurance coverage of telemedicine are paramount for a wider uptake by the patients [27].

## Data Availability

All data and material collected are presented in the manuscript. Clarification on any matter can be made through the corresponding author.
